# Analysis and Comparison of Gut Microbiome in Young Detection Dogs

**DOI:** 10.3389/fmicb.2022.872230

**Published:** 2022-04-19

**Authors:** Zongjie Li, Qing Sun, Yuhao Li, Zhixin Guan, Jianchao Wei, Beibei Li, Ke Liu, Donghua Shao, Rongsheng Mi, Haixia Liu, Yafeng Qiu, Zhiyong Ma

**Affiliations:** ^1^Shanghai Veterinary Research Institute, Chinese Academy of Agricultural Science, Shanghai, China; ^2^Beijing Huayuan Biotechnology Research Institute, Beijing, China

**Keywords:** detection dog, gut microbiota, olfactory function, Springer Spaniel, Labrador Retriever, German Shepherd

## Abstract

The detection dogs are well-known for their excellent capabilities to sense different kinds of smells, which can play an important role in completing various searching and rescuing missions. The recent studies have demonstrated that the excellent olfactory function of detection dogs might be related with the gut microbes *via* the bidirectional communications between the gastrointestinal tract and the brain. In this study, the gut microbial communities of three types of breeds of detection dogs (Springer Spaniel, Labrador Retriever, and German Shepherd) were studied and compared. The results revealed that the richness and the diversity of gut microbiome German Shepherd dogs were significantly higher than the Labrador Retriever dogs and the Springer Spaniel dogs. At the phylum level, the most predominant gut microbial communities of the detection dogs were comprised of Fusobacteriota, Bacteroidetes, Firmicutes, Proteobacteria, Campilobacterota, and Actinobacteriota. At the genus level the most predominant gut microbial communities were comprised of *Fusobacterium, Megamonas, Prevotella, Alloprevotella, Bacteroides, Haemophilus, Anaerobiospirillum, Helicobacter, Megasphaera, Peptoclostridium, Phascolarctobacterium*, and *Streptococcus*. However, the gut microbial communities of the three dogs group were also obviously different. The mean relative abundance of *Fusobacterium, Prevotella, Alloprevotella, Megamonas, Bacteroides*, and *Phascolarctobacterium* presented significant differences in the three groups. According to the portraits and characteristics of the gut microbiome in young detection dogs, multiple kinds of nutritional interventions could be applied to manipulate the gut microbiota, with the aim of improving the health states and the olfactory performances.

## Introduction

The detection canines have been widely used for executing various kinds of searches and rescue missions, and their excellent performances in identifying discriminations are also have intimate relations with the olfactory capabilities to detect different odors and chemical compounds (Jenkins et al., 2018). Current studies have revealed that the physical condition and olfaction performance of the working canines were closely related with the symbiotic microbiome, including the nasal, oral and gut microbiota (Suchodolski et al., 2009; Isaiah et al., 2017a; Emilie et al., 2021). Alterations of the gut microbiota induced by dietary structure, drug administration, and living environments can all influence the health conditions and olfactory capabilities of the detection canines (Jenkins et al., 2016; Herstad et al., 2017; Essler et al., 2019). Therefore, manipulations of the gut microbiota through multiple strategies can be applied as novel interventions to improve the odor detecting properties of working dogs.

During the 30,000 years of domesticating process, the companion dogs gradually adapted to the human living environments and appeared to be interested in the human social cues (Wu et al., 2017). At the same time, the richness and diversity of the companion dogs' gut microbiome also changed significantly to adapt the domesticated living environment, while the hierarchical clustering of gastrointestinal metagenomes has proved the phylogenetic and metabolic similarities between the dogs and humans (Swanson et al., 2011). The high-throughput sequencing technique research data also proved that the gut microbiome of the companion dogs shared certain similarities with their owners (Deng and Swanson, 2015; Wang et al., 2022). Therefore, further study is required to identify the regulating effects of genetics, environments, and domestications on the composition of the canine microbiome, and to evaluate its role in canine immune function and gastrointestinal health.

In fact, the search and rescue performances of the detection dogs can be improved by extensive training and good dog-handler relationships (Diverio et al., 2016). Because the development of animal behavioral profiles can be powerfully influenced by the social experiences in the early life phases, the capabilities training of the detection dog to sense different smells can be started in the growing process (Sachser et al., 2011). The potential odor recognizing abilities of detection dogs can be improved by well-established training practice using the explosive samples or other organic chemicals (Lucia and David, 2014). By comparing the brain sizes between the ancestral species and the domesticated relatives, artificial domestication and social adaption could influence the brain function and behavioral development (Kruska, 2005). The bidirectional communications between the gut and the brain can be realized through the vagus nerve, the neuroendocrine pathways, and the bacteria-derived metabolites, and the brain function and the behavioral profiles can be influenced by the microbiota–gut–brain axis (Sandhu et al., 2017). Therefore, the olfactory performances of detection dogs might have certain relations with their unique gut microbial communities, and their physiological and behavioral conditions could be changed by gut microbiome alteration (Hooda et al., 2012).

Diet structure is commonly regarded as a critical influencing factor on the dog gut microbiota, which can produce important effects for gut health and overall well-being (Herstad et al., 2017). When the canine diet was changed from commercial dry food to mildly cooked diet (such as boiled minced beef), the microbial communities of the gut microbiota and the fecal metabolism profile were also changed (Tanprasertsuk et al., 2021). Compared with the commercial extruded diet, the administration of the raw-based diet supplemented with vegetable foods could promote the balance of dog–gut–microbial ecosystem and improve the gut function of healthy dogs (Sandri et al., 2017). Bones and raw food diets contained a high amount of meat, offal, and raw meaty bones, which combined with small amounts of vegetables and fruits and different sorts of oil and supplements. The previous study proved that the gut microbial communities and metabolome were significant different between the bones and raw food diets fed dogs and the commercially fed dogs (Schmidt et al., 2018). However, other studies revealed that a high protein diet could increase the abundance of butyrate-producing bacteria in Beagles, and had a greater impact on the microbial communities of the obese dogs (Xu et al., 2017). Therefore, the regulating role of diet consumption on the gut microbiome should also not be neglected besides the genetic portraits.

In the early life period, the acquisition of gut microbiome in young detection canines can be influenced by many factors, and it is a critical phase to establish the well-balanced microbial community and the maturated and developed immune system. The probiotics interventions targeting gut microbiota can confer health benefits for the host and forbid the invasion of foreign pathogens into the gastrointestinal tract. Fecal microbiota transplantation (FMT) is also recognized as an effective treatment for recurrent *Clostridioides difficile* infection; therefore, FMT can also provide valuable benefits for dogs with acute and chronic digestive diseases (Chaitman and Gaschen, 2021). Moreover, supplementations of probiotics can also prevent and treat the allergy and acute gastroenteritis of the companion dogs (Grześkowiak et al., 2015). In this study, the extraordinary compositions of gut microbiota in different breed types of young detection dogs were investigated and compared, and their possible functions, the gastrointestinal tract health, and the olfactory properties were predicted and analyzed.

## Materials and Methods

### Animals and Diet

**Figure 1:**
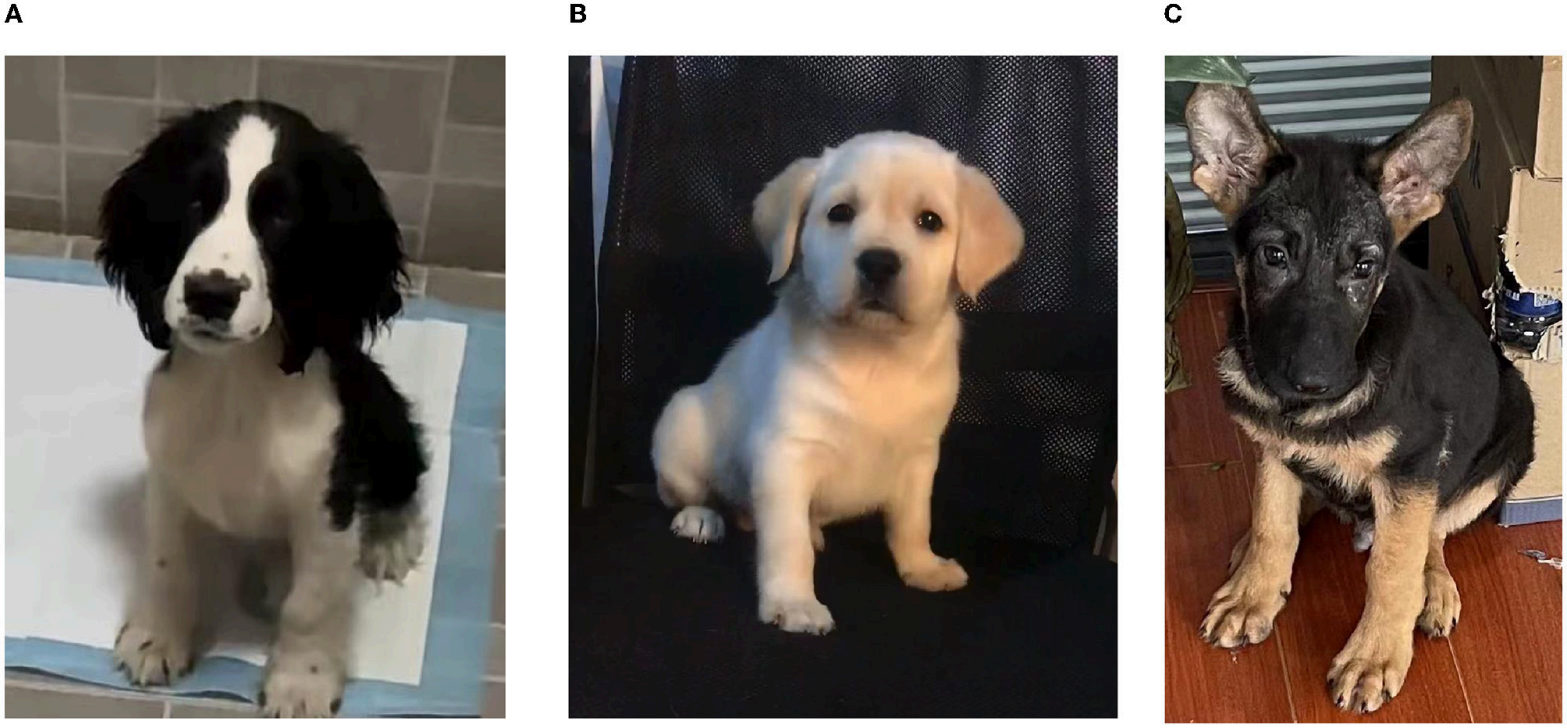
Three breed types of detection dogs recruited in this study. **(A)** Springer Spaniel dog **(B)** Labrador retriever dog **(C)** German Shepherd dog.

A total of 14 healthy dogs (five females and nine males) belonging to three different kinds of breed were recruited at the Shanghai Jialiang Working Dog Center (Figure 1). The participant dogs were categorized into three groups, including German Shepherd dogs (group D, *n* = 5), Labrador Retriever dogs (group L, *n* = 5), and Springer Spaniel dogs (group S, *n* = 4; Table 1).

**Table 1:** Basal characteristics of the enrolled detection dogs.

**Breed groups**	**Sex (female/ male)**	**Age (months)**	**Body weight (kg)**
S	2/2	2.3	3.1
L	2/3	2.5	5.5
D	1/4	2.8	7.3

All the dogs were fed with a commercial diet (Fubei PetCare, Shanghai, CN; Table 2). All the dogs were housed under the same environment and had free access to drink water, and all the dogs were maintained routinely by a professional breeder without any additional treatment. None of the enrolled dogs presented a history of medication, neutralization, or diarrhea before and after the experiment. All experimental procedures in this study were approved by the Committee for Accreditation of Laboratory Animal Care and the Guideline for the Care and Use of Laboratory Animals of Shanghai Veterinary Research Institute, Chinese Academy of Agricultural Science (approval number: 20210615).

**Table 2:** Primary ingredients and nutritional composition of the utilized diets.

**Ingredients**	**Nutrient composition**
Crude protein	26%
Crude fat	12%
Crude fiber	5%
Crude Ash	10%
Calcium	1.1%
Phosphorus	0.9%
Tysine	1.0%
Chloride	0.5%
Moisture	10%
Energy	442 kcal/100 g

The dogs were maintained open during the sampling process, and sterile swabs (Copan^®^, FLOQSwabs ™, 553C, Brescia, Italy) were introduced through the anus up to one third of the distal rectum. Three gentle complete circular movements were used to brush the mucosa before the withdrawal of the swab (Bell et al., 2020). Then the tops of the fecal swabs were cut and stored in sterile cryotubes at −80°C.

### DNA Isolation

The collected fecal swabs were aliquoted into a sterile 2 ml tube containing 250 μl of 0.1 mm zirconia-silica beads, and then the samples were homogenized for a duration of 1 min at a speed of 5 m/s (Guard et al., 2015). Then the DNA was extracted with the QIAamp Mini Kit (Qiagen, Hilden, Germany) following the manufacturer's recommendations. The extracted genomic DNA quality was verified by agarose gel electrophoresis, and the total DNA concentration was measured by optical density ratio at 260 nm/280 nm using a spectrophotometry reader (NanoDrop, Thermo Scientific). The extracted DNA was stored at −20°C for further analyses.

### 16S rDNA Amplicons Sequencing

The V3/V4 hypervariable regions of the bacterial 16S rRNA gene were amplificated using the following primers: 341F(5′-CTACGGGNGGCWGCAG-3′) and 805R (5′- GACTACHVGGGTATCTAATCC−3′). All the 16S rRNA gene amplicons were used for constructing DNA libraries and were sequenced using the Illumina NovaSeq PE250 platform (You and Kim, 2021).

### Microbial Community Analysis

The bioinformatic analysis was performed using the quantitative insights into microbial ecology (QIIME) package (version 2). The sequencing results were firstly converted to FASTQ files based on the Illumina index sequences, and then the adapter sequences were trimmed using FASTP and the overlapping regions were demultiplexed. After removing the low-quality sequences, the remaining reads were clustered into operational taxonomic units (OTUs) with 97% sequence similarity. The 16S rRNA gene sequences were aligned by PyNAST and clustered under 100% sequence identity by UCLUST, and the microbial community analysis of the observed OTUs was performed based on the SILVA database (Emilie et al., 2021; You and Kim, 2021). The sequence biodiversity and richness were evaluated by the rank–abundance curves, and the Venn diagram was calculated to assess the microbiota structure in different samples. The alpha diversity analysis was calculated by the Chao1, ACE, Shannon and Simpson index, and the richness and diversity of the microbial community within different groups were measured using QIIME 2. The beta diversity was evaluated using the visualized principal coordinate analysis (PCoA) based on the unweighted UniFrac distances, and the microbial compositions between different groups were estimated and compared (Xu et al., 2019). The significant differences in OTUs abundance among the three groups were analyzed by non-parametric Kruskal–Wallis test. The PICRUSt package were used to predict the contribution of bacterial community genes for potential function through the EggNOG (evolutionary genealogy of genes: Non-supervised Orthologous Groups) database (http://eggnog.embl.de/). All other analyses and visualizations were performed with R software version 3.0.1 and the boxplot package (Suchodolski et al., 2012; Minamoto et al., 2015; Zhang C. et al., 2015; Isaiah et al., 2017b; Li et al., 2021).

### Statistical Analysis

In the comparison of microbial diversity index and relative abundance, the Kruskal–Wallis test or Wilcoxon rank sum test were used, and *p* < 0.05 was determined statistically significant for other statistical analyses.

### Sequence Data Accession Numbers

The 16S rRNA gene sequencing data reported in this study have been submitted to the GenBank Sequence Read Archive database (accession numbers of PRJNA803605).

## Results

### Quality Control of the Sequenced Data and OTUs Analysis

The raw data obtained from the sequencing instrument was demultiplexed and quality filtered using QIIME2. Firstly, a total of 4,829,067 filtered reads were obtained from the 5,104,464 raw reads, and then the denoised analysis was performed by discarding the ambiguous reads and the primer matched barcoded reads were merged. Finally, a total of 2,790,859 clean reads were assembled into qualified sequences and used for further analysis (Table 3).

**Table 3:** Summary of sequencing data in the dog gut microbiota.

**Amplified region**	**Input**	**Filtered**	**Denoised**	**Merges**	**Non-chimera**
341F_805R	5104464	4829067	4740078	4147147	2790859

**Figure 2:**
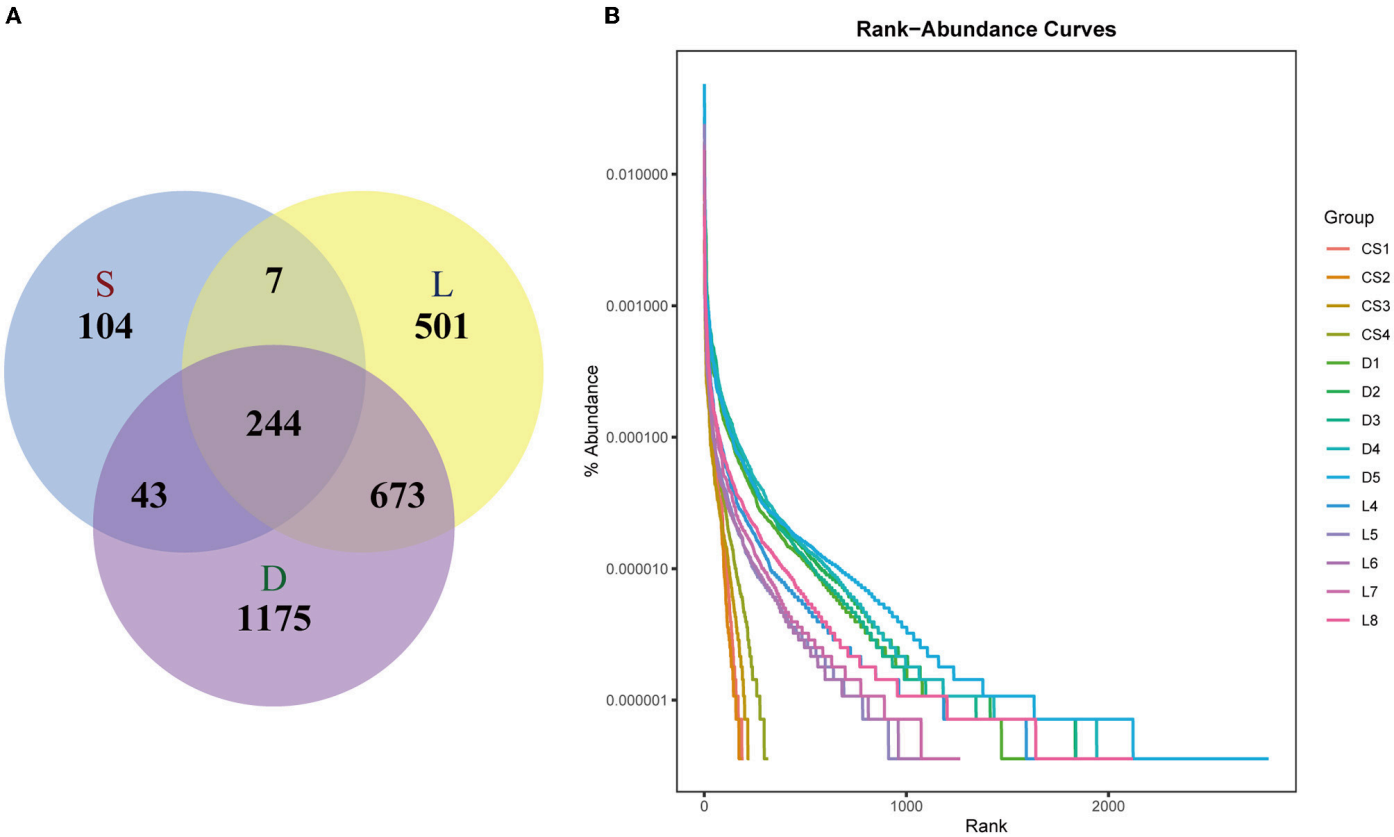
Venn diagram **(A)** and the rank–abundance curves **(B)**. There were 244 shared OTUs among the three groups, wherein the OTUs number of German Shepherd group (2,135) was the highest, and then the OTUs number of Labrador retriever group (1,425) was much higher than that of the Springer Spaniel group (398). The vertical axis of the rank–abundance curves showed the percent of OTUs after sampling, while the horizontal axis showed the number of sequences, and the tail length of rank–abundance curves revealed the bacterial community richness.

The high-quality non-chimera sequences were clustered into OTUs with at least 97% sequence identity. As showed in Figure 2A, there were 244 shared OTUs among the three groups; however, each group also had its corresponding unique OTUs. The Venn diagrams demonstrated that the OTUs numbers of the German Shepherd group (2,135) was the highest, which was much higher than those of the Labrador Retriever group (1,425) and the Springer Spaniel group (398). The comparison results of OTUs number indicated that the gut microbial diversity of German Shepherd dogs was much higher than the Labrador Retriever dogs and the Springer Spaniel dogs. The tail lengths of rank–abundance curves at the horizontal axis showed the bacterial community richness of the German Shepherd group was much higher than those of the Labrador Retriever group and the Springer Spaniel group (Figure 2B).

### Diversity Analysis of Gut Microbial Communities

**Figure 3:**
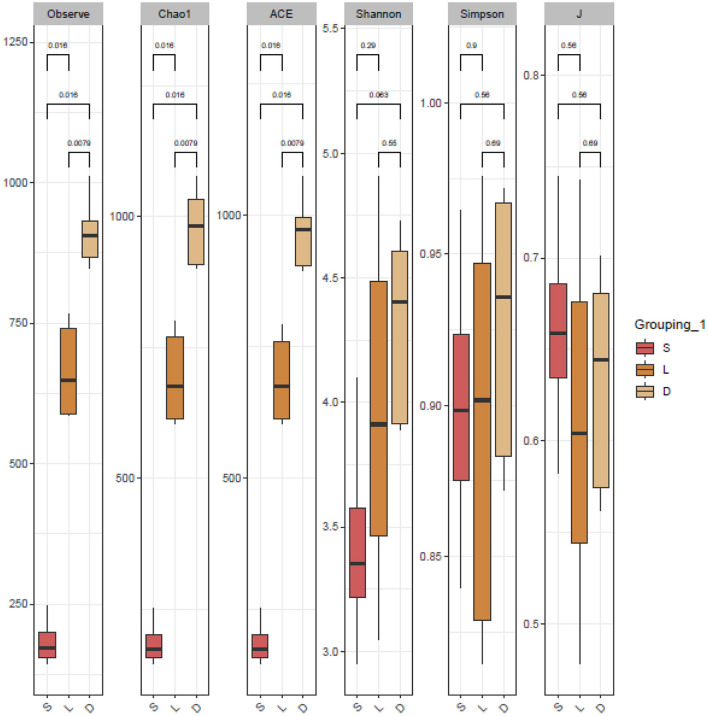
Alpha diversity analysis of the dog gut microbial community. The richness and diversity estimators of observe, Chao1, ACE, Shannon, Simpson, and J indices were calculated, respectively. The observe, ACE, and Chao1 indices indicated that the bacterial richness of German Shepherd group was much higher than the other two groups, and Shannon and Simpson indices revealed that bacterial community diversity of German Shepherd was the highest.

**Figure 4:**
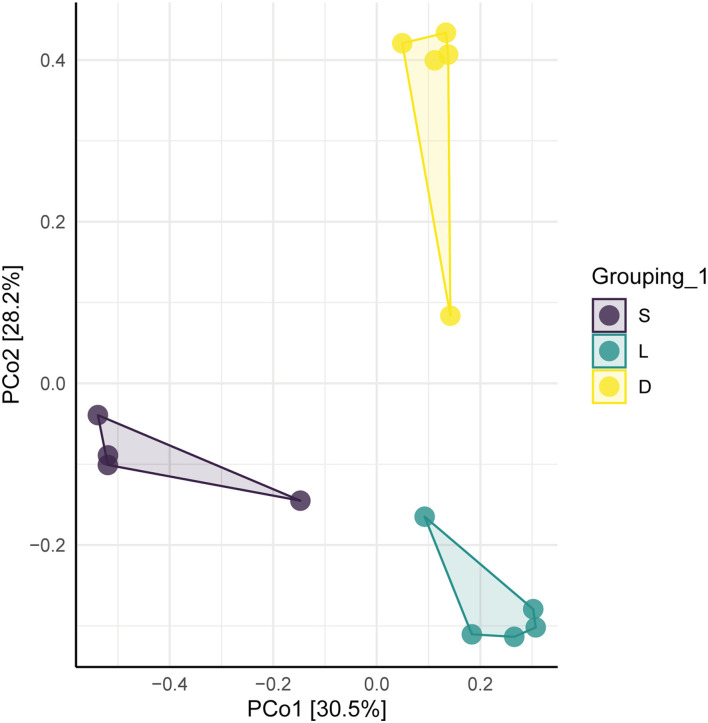
Plot of PcoA of the dog gut microbial community. The three different groups were segregated into different community clusters.

The clustered OTUs were analyzed using the RDP Classifier against the SILVA database with a confidence threshold of 70%. The alpha diversity analysis of the gut microbial community was evaluated by the observe, Chao1, ACE, Shannon, Simpson, and J indices (Figure 3). The calculated observe, ACE, and Chao1 indices indicated that the bacterial richness of the German Shepherd group was significantly higher than the Labrador Retriever group and the Springer Spaniel group. At the same time, the Shannon and Simpson indices revealed that the gut bacterial diversity of the German Shepherd group was much higher than the other two groups. The beta diversity analysis was estimated by the PCoA based on the unweighted UniFrac distance, and the clustering results demonstrated that the three different groups were segregated into different clusters (Figure 4).

### Microbial Compositions of the Gut Microbiota

**Figure 5:**
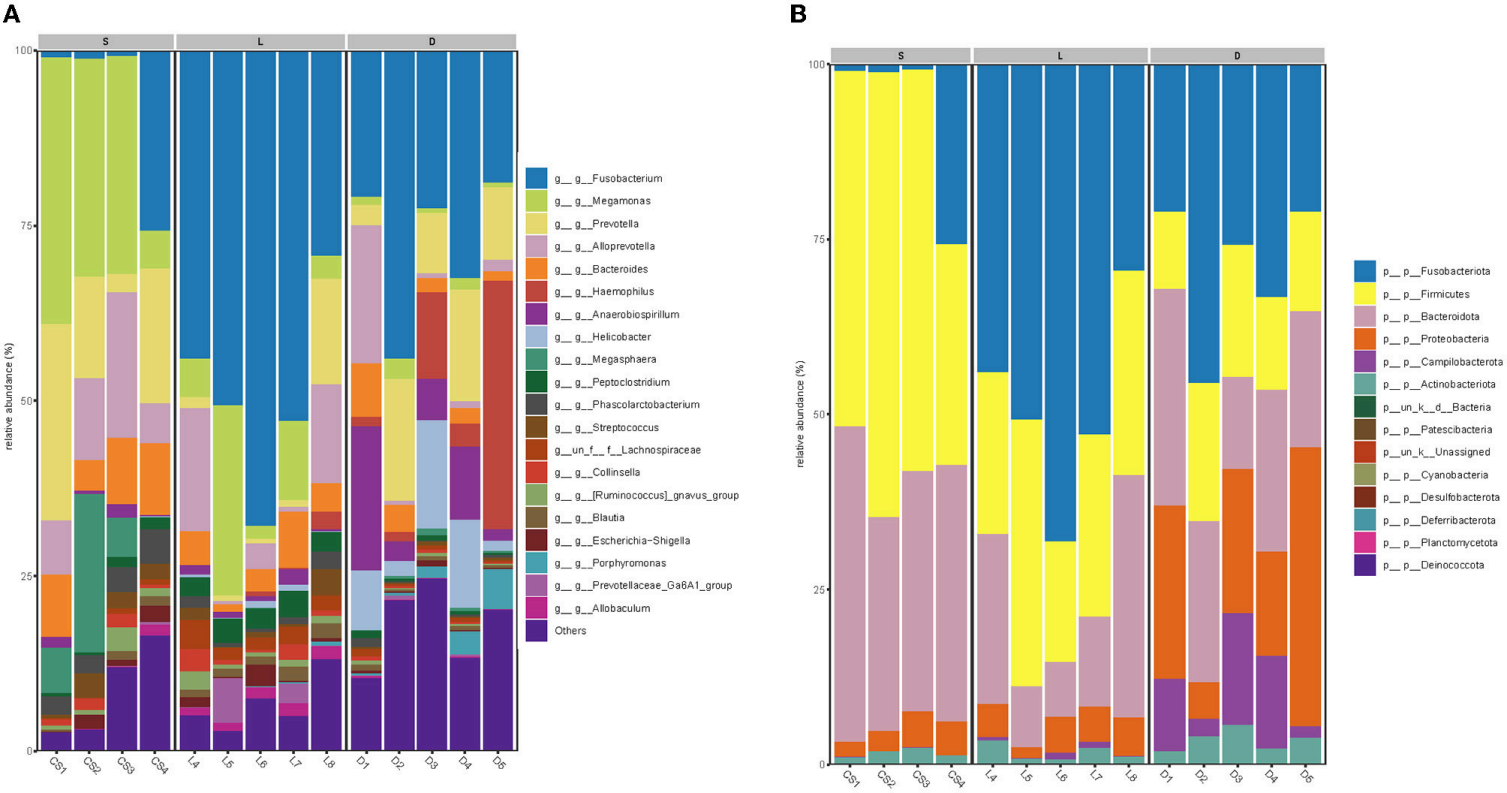
The compositions of dog gut bacterial community at the phylum **(A)** and genus **(B)** levels. Less than 1% abundance of bacterial taxa at the phyla or genus levels was merged into others, and the microbial communities of the three groups differed obviously.

The taxonomic composition of the detection dogs' gut microbial communities was analyzed at the phylum level and the genus level, respectively (Figures 5A,B). Across all the sequenced samples, the most predominant microbial communities were showed in Table 4. However, the microbial communities of the three groups at the phylum level differed apparently. The most predominant gut microbial communities at the phylum level were comprised of Fusobacteriota (29.81%), Bacteroidetes (29.78%), Firmicutes (24.54%), Proteobacteria (10.17%), Campilobacterota (3.29%), and Actinobacteriota (2.27%; showed in Figure 5A). As showed in Figure 5B, the most predominant gut microbial communities at the genus level were comprised of *Fusobacterium* (29.19%), *Megamonas* (11.53%), *Prevotella* (9.85%), *Alloprevotella* (7.54%), *Bacteroides* (5.09%), *Haemophilus* (4.04%), *Anaerobiospirillum* (3.63%), *Helicobacter* (3.01%), *Megasphaera* (2.65%), *Peptoclostridium* (1.63%), *Phascolarctobacterium* (1.57%), and *Streptococcus* (1.22%). The gut microbial communities of the three group of detection dogs at the genus level were also obviously different.

**Table 4:** The predominant taxonomic profiles of the dog gut microbiota.

**Sample group**	**Taxonomic level**	**Phyla**	**Relative abundance (%)**
	Phylum	Fusobacteriota	29.81
		Firmicutes	29.78
		Bacteroidetes	24.54
		Proteobacteria	10.17
		Campilobacterota	3.29
		Actinobacteriota	2.27
	Genus	*Fusobacterium*	29.19
		*Megamonas*	11.53
		*Prevotella*	9.85
		*Alloprevotella*	7.54
		*Bacteroides*	5.09
		*Haemophilus*	4.04
		*Anaerobiospirillum*	3.63
		*Helicobacter*	3.01
		*Megasphaera*	2.65
		*Peptoclostridium*	1.63
		*Phascolarctobacterium*	1.57
		*Streptococcus*	1.22

### Comparisons of the Gut Microbial Communities

**Figure 6:**
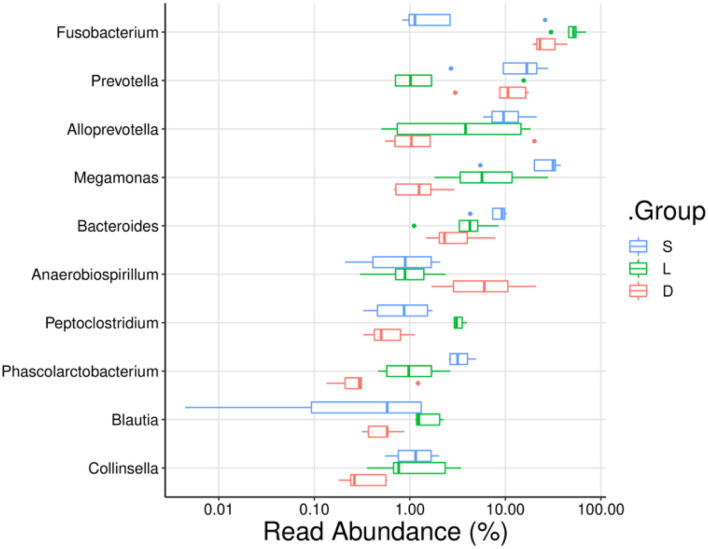
Comparisons of the relative abundance of dog gut microbiota at the genus level. The ordinate indicated the bacterial name at genus levels, and the abscissa indicates the abundance percentage values of the samples.

**Figure 7:**
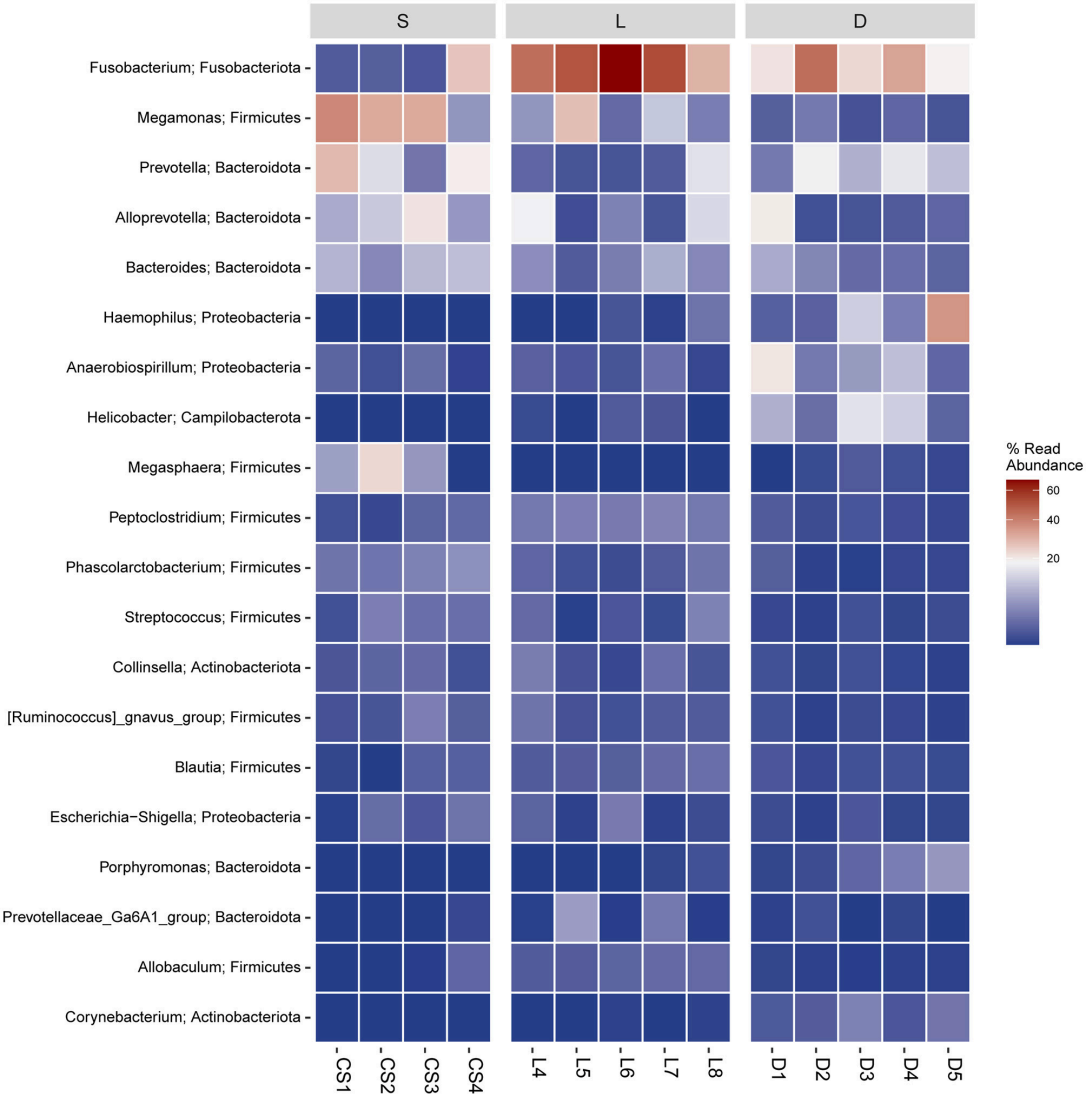
Heatmap of hierarchy cluster results for the abundance of genus. The genus names of the OTUs are shown on the left, and the different colors of the spots indicated the normalized and log-transformed relative abundance.

To compare the mean percentage of the predominant genera among the three groups, the significant differences in the relative abundance of detection dogs' gut microbiota were analyzed using the Kruskal–Wallis test. The percentages of *Fusobacterium, Prevotella, Alloprevotella, Megamonas, Bacteroides*, and *Phascolarctobacterium* presented significant differences in the three groups (Figures 6, 7). In detail, the percentage of *Fusobacterium* in the Labrador Retriever group (48.39%) was the highest, which was much higher than those of the German Shepherd group (27.60%) and the Springer Spaniel group (7.20%). The percentage of *Prevotella* in the Springer Spaniel group (16.06%) was the highest, which was higher than those of the German Shepherd group (10.99%) and the Labrador Retriever group (3.76%). Similarly, the percentage of *Alloprevotella* in the Springer Spaniel group (11.50%) was also the highest, which was higher than those of the Labrador Retriever group (7.23%) and the German Shepherd group (4.68%). Moreover, the percentage of *Megamonas* in the Springer Spaniel group (26.41%) was also the highest, which was higher than those of the Labrador Retriever group (9.74%) and the German Shepherd group (1.41%). The relative abundances of *Bacteroides* and *Phascolarctobacterium* in the Springer Spaniel group were also higher than the other two groups (*p* < 0.01). However, the relative abundance of *Lactobacillus* in the German Shepherd group (0.87%) was much higher than those in the Springer Spaniel group (0.61%) and the Labrador Retriever group (0.12%). Due to the reason that all these three groups of dogs lived in a similar environment and consumed the same diet, the marked differences in gut microbial communities might be associated with their unique breed types.

### PICRUSt Functional Prediction

**Figure 8:**
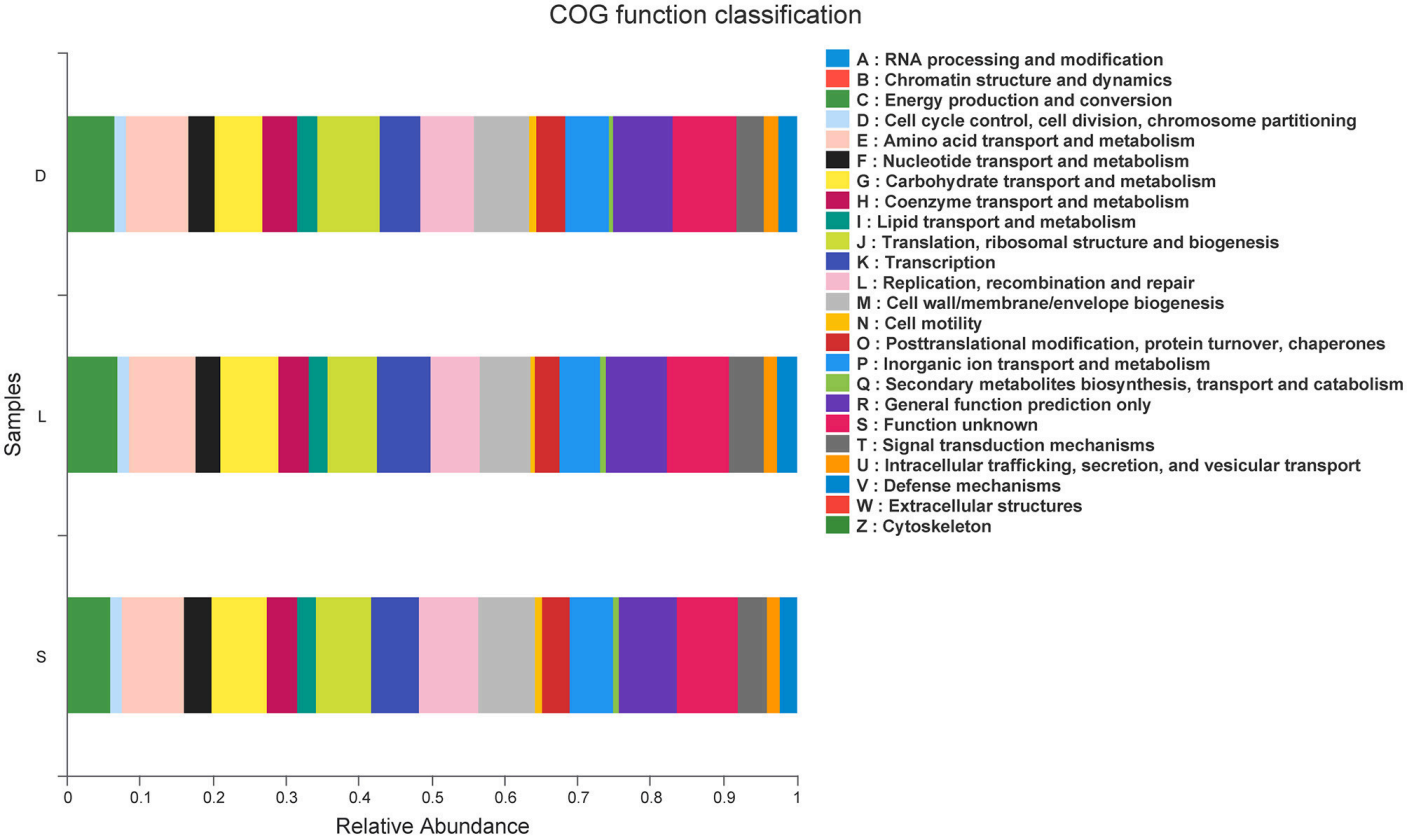
PICRUSt functional prediction was performed using EggNOG database, and pathways related to dog gastrointestinal diseases were identified.

The predicted functions were calculated based on PICRUSt in EggNOG database, and a total of 22 pathways related to the dog gastrointestinal tract diseases were identified (Figure 8). The predicted microbial genes related to the defense mechanisms in the German Shepherd group (3,125,562) were much higher than those in the Labrador Retriever group (2,135,376) and the Springer Spaniel group (859,726), indicated that the German Shepherd dog might had a stronger immunity to fight against the gastrointestinal tract infectious diseases. Moreover, the predicted microbial genes related to the carbohydrate transport and metabolism in the German Shepherd group (8,747,138) were also much higher than those in the Labrador Retriever group (6,763,335) and the Springer Spaniel group (2,904,780), which meant that the German Shepherd dog had a stronger carbohydrate metabolic ability. Correspondingly, the relative abundances of lipid transport and metabolism, amino acid transport and metabolism, and energy production and conversion microbial genes in the German Shepherd group were also much higher than the other two groups. In all, the predicted functions of microbial genes demonstrated that the gut microbiome of the German Shepherd dog might provide more effective energy supply and stronger immune protection.

## Discussion

During the long term of mammal animal evolutionary process, the human artificial domestication and hybridization on companion dogs had generated obvious morphology differences and behavioral responses when compared with their wild ancestors. The typical changes in the appearance characteristics (such as curly tails, floppy ears, white patches, and shorter muzzles) and behavioral profiles (such as social behaviors, cognitive abilities, and emotional responses) of domestic canines might have close relations with the physiological shifts and genetic alterations (Kaiser et al., 2015; Lesch et al., 2022). In this study, the extraordinary compositions of gut microbiota in different breed types of young detection dogs were investigated and compared, and the possible relations between their gut microbiota and working performances were also analyzed and discussed.

The gut microbial composition of detection dogs might be influenced by diet structure, living environment, exercise management, and other impacting factors. However, the microbial species differed apparently upon the different canine breeds according to the results shown in this study. Venn diagrams showed that the OTUs number of German Shepherd was the highest, which meant that the gut microbial diversity of German Shepherd dogs was much higher than the Labrador Retriever dogs and the Springer Spaniel dogs (showed in Figure 2A). The longer rank–abundance curves tails of the German Shepherd dogs at the horizontal axis indicated the bacterial community richness of German Shepherd dogs was also much higher than the other two groups (Figure 2B). For the alpha diversity analysis, the calculated observe, ACE, and Chao1 indices indicated that the bacterial richness of German Shepherd dogs was the highest among the three groups, while the Shannon and Simpson indices revealed that the diversity of German Shepherd dogs' gut microbiota was also higher than the Labrador Retriever group and the Springer Spaniel group (Figure 3). For beta diversity analysis, the PCoA based on the unweighted UniFrac distance demonstrated that the gut microbes of the three groups were clustered into different communities (Figure 4). Therefore, the current research data revealed that the richness and diversity of gut microbiota in different breeds of detection dogs differed obviously, which suggested that the genetic portrait might be a major determining factor on the gut microbiota.

According to the previous studies, the gut microbiome played an important role in maintaining the host health state, for the reason that the gut microbiome could educate the immune system, regulate the energy metabolism, and fight against the invading pathogens (Mondo et al., 2019; Pilla and Suchodolski, 2020). Therefore, the multiple physiological functions of certain members in the gut microbiome are worthy to be further studied. In the present study, the taxonomic compositions of the gut microbial communities were separately analyzed at the phylum level and the genus level (Figures 5A,B). At the genus level, the members of *Phascolarctobacterium, Blautia, Ruminococcus*, and *Coprococcus* were identified in the gastrointestinal tract of detection dogs. These four genera are well-known for the abilities of fermenting carbohydrate to produce short-chain fatty acids (SCFAs), which can help the host to maintain the immune homeostasis and regulate the energy metabolism (Zhang J. et al., 2015). Results also demonstrated that the gut microbial communities of the three group of detection dogs were obviously different, and then the most predominant gut microbial taxa were investigated and compared. The mean relative abundance of *Fusobacterium, Prevotella, Alloprevotella, Megamonas, Bacteroides*, and *Phascolarctobacterium* presented significant differences in the three groups (Figures 6, 7). In detail, the percentage of *Fusobacterium* in the Labrador Retriever group was much higher than those of the German Shepherd group and the Springer Spaniel group. However, the percentage of *Prevotella, Alloprevotella, Megamonas, Bacteroides*, and *Phascolarctobacterium* in the Springer Spaniel group was higher than those of the German Shepherd group and the Labrador. Interestingly, the relative abundance of *Lactobacillus* in the German Shepherd group (0.87%) was found to be much higher than the Springer Spaniel group (0.61%) and the Labrador Retriever group (0.12%). The *Lactobacillus* might generate multiple kinds of beneficial metabolites to protect the gastrointestinal tract function (Hang et al., 2013). Therefore, the remarkable differences of the gut microbiome in the three groups might be associated with their corresponding genetic backgrounds, and the characteristics of the gut microbiome might reveal the breed portraits of the detection dogs.

The canine gastrointestinal microbes had important roles for the nutritional, immunological, and physiologic functions, while the microbiome dysbiosis caused by various reasons could induce canine chronic diarrhea and inflammatory bowel diseases (Hooda et al., 2012; Omori et al., 2017). The predicted PICRUSt functions *via* EggNOG database revealed the immune regulating roles of the gut microbiome. The predicted microbial genes related to the defense mechanisms, carbohydrate transport and metabolism, lipid transport and metabolism, amino acid transport and metabolism, and energy production and conversion in the German Shepherd group were found to be higher than the Labrador Retriever group and the Springer Spaniel group, which meant that the gut microbiome of the German Shepherd dog could provide more effective energy supply and stronger protection against the gastrointestinal tract diseases. The most important characteristics of the detection dogs are their excellent capabilities to sense different kinds of smells, and their olfaction could be impacted by gut microbes through the bidirectional communications between the gastrointestinal tract and brain. However, dietary fiber, prebiotics, probiotics, and other dietary interventions could be applied to regulate the canine gut microbiome and improve the health indices (Bell et al., 2020). Therefore, novel techniques to manipulate the gastrointestinal microbiota could be explored to improve the olfactory performance of working canines.

## Data Availability Statement

The original contributions presented in the study are publicly available. This data can be found at: https://www.ncbi.nlm.nih.gov/search/all/?term=PRJNA803605.

## Ethics Statement

The animal study was reviewed and approved by Committee for Accreditation of Laboratory Animal Care and the Guideline for the Care and Use of Laboratory Animals of Shanghai Veterinary Research Institute.

## Author Contributions

All authors researched data for the article, made substantial contribution to discussion of content, and wrote, reviewed and edited the manuscript before submission. All authors contributed to the article and approved the submitted version.

## Funding

This work was supported by the Shanghai Rising-Star Program (19QA1411200) and the Chinese National Natural Science Foundation Grant (31672606).

## Conflict of Interest

The authors declare that the research was conducted in the absence of any commercial or financial relationships that could be construed as a potential conflict of interest.

## Publisher's Note

All claims expressed in this article are solely those of the authors and do not necessarily represent those of their affiliated organizations, or those of the publisher, the editors and the reviewers. Any product that may be evaluated in this article, or claim that may be made by its manufacturer, is not guaranteed or endorsed by the publisher.
